# Eelgrass (*Zostera marina*) Food Web Structure in Different Environmental Settings

**DOI:** 10.1371/journal.pone.0146479

**Published:** 2016-01-11

**Authors:** Jonas Thormar, Harald Hasler-Sheetal, Susanne Baden, Christoffer Boström, Kevin Kuhlmann Clausen, Dorte Krause-Jensen, Birgit Olesen, Jonas Ribergaard Rasmussen, Carl Johan Svensson, Marianne Holmer

**Affiliations:** 1 Department of Biosciences, University of Oslo, Oslo, Norway; 2 Department of Biology, University of Southern Denmark, Odense, Denmark; 3 Nordic Center for Earth Evolution (NordCEE), University of Southern Denmark, Odense, Denmark; 4 Department of Biology and Environmental Sciences, University of Gothenburg, Fiskebäckskil, Sweden; 5 Department of Biosciences, Environmental and Marine Biology, Åbo Akademi University, Åbo, Finland; 6 Department of Bioscience, Aarhus University, Kalø, Denmark; 7 Department of Bioscience, Aarhus University, Silkeborg, Denmark; 8 Department of Bioscience, Aarhus University, Aarhus C, Denmark; 9 Department of Infectious Diseases, Institute of Biomedicine, University of Gothenburg, Göteborg, Sweden; College of Charleston, UNITED STATES

## Abstract

This study compares the structure of eelgrass (*Zostera marina* L.) meadows and associated food webs in two eelgrass habitats in Denmark, differing in exposure, connection to the open sea, nutrient enrichment and water transparency. Meadow structure strongly reflected the environmental conditions in each habitat. The eutrophicated, protected site had higher biomass of filamentous algae, lower eelgrass biomass and shoot density, longer and narrower leaves, and higher above to below ground biomass ratio compared to the less nutrient-enriched and more exposed site. The faunal community composition and food web structure also differed markedly between sites with the eutrophicated, enclosed site having higher biomass of consumers and less complex food web. These relationships resulted in a column shaped biomass distribution of the consumers at the eutrophicated site whereas the less nutrient-rich site showed a pyramidal biomass distribution of consumers coupled with a more diverse consumer community. The differences in meadow and food web structure of the two seagrass habitats, suggest how physical setting may shape ecosystem response and resilience to anthropogenic pressure. We encourage larger, replicated studies to further disentangle the effects of different environmental variables on seagrass food web structure.

## Introduction

In most coastal areas, seagrass meadows are an integrated and important part of the shallow water food web. As engineering species with high primary production, large surface area and a well-developed below ground system, seagrasses provide ecosystem functions and services, including carbon sequestration, nutrient binding and stabilization of coastal sediments [1]. Moreover, and equally important, they form an important habitat for associated fish and invertebrate species, including commercially valuable ones, by providing food and substrate, as well as shelter, nursery and feeding areas [2]. This range of central ecosystem services also renders seagrasses socio-economically important [3–5].

Human activities in the marine environment have been shown to impact coastal ecosystems [6] and responses may depend on the local environmental regime through physical-biological couplings [7]. Recognizing and understanding ecosystem structure and function under different environmental settings is therefore essential for our predictive ability of how ecosystem health and services can be sustained [8]. Seagrass meadows worldwide have experienced losses in particular due to shading caused by drifting algae, planktonic algal blooms and suspended material in the water column [9–14]. Bottom-up processes causing blooms of overgrowing planktonic or filamentous algae have long been seen as the major problem, but these processes may act in concert with top-down forces, such as the overfishing of large predatory fish [15–18]. In both processes mesograzers are known to play a key role, both as prey for predatory fish and efficient consumers of algae [19, 20]. Recognizing the strength and direction of different trophic links in seagrass meadows may provide insight into the function and resilience of coastal ecosystems in general.

Shifts in primary producer structure towards ephemeral algae and phytoplankton dominance in response to eutrophication are well documented in marine ecosystems [7, 8, 21, 22]. However, there is limited information on the subsequent response of the consumer food webs to such changes [12, 23]. This information suggests that decreased fish diversity and decapod and fish biomass is linked to increased nitrogen load in seagrass ecosystems [12]. Furthermore, network analysis has shown that trophic structure may be affected, resulting in simplified food chains and vertically compacted biomass pyramids with an increased fraction of herbivores and intermediate predators, and lowered robustness towards species losses [23].

Physical settings may also influence the community structure and biological responses to eutrophication [7]. While the effect of physical exposure from wind, waves or currents (hereafter exposure) on seagrass meadow structure is well studied (e.g. [24–27]), we found only one study [27] on the interactive effects of exposure and eutrophication on seagrass demography. A positive correlation between wind exposure and biomass has been found for epifaunal bivalves and barnacles [28], while a range of decapods and small fish appear to be negatively affected [29]. In intertidal seagrass meadows, exposure may also reduce the abundance of gastropod grazers which allows for increased growth of epiphytic algae [30]. However, to our knowledge, there is no comprehensive study on the effect of exposure, or its interactive effect with eutrophication, on the entire food web in a seagrass meadow. Another setting is spatial and hydrological conditions that may limit the movement and dispersal of organisms between habitats and it is widely recognized that connectivity between seagrass meadows and surrounding habitats influence the faunal abundances and dynamics of tropical marine habitats (e.g. [31]). Connectivity with other habitats can also be important in maintaining biodiversity [32] and thus potentially community stability [33, 34]. However seagrass studies have found little effect of patch or landscape connectivity on species diversity (see [35]), and dispersal may even have a destabilizing effect [36]. The knowledge of potential effects of connectivity level on temperate seagrass meadows and entire food webs is scarce [35], but theory suggests shortened food chains and reduced food web stability at low connectivity [37].

In systems with high biomass of primary producers, such as vegetated benthic ecosystems, theory suggests that prolonged stress induces shorter and/or simpler food chains due to reduced energy flow to higher trophic levels and higher sensitivity of predators to stress [38–40]. A predictable sign of human impact is a change in the trophic structure [39, 41, 42]. This can be exemplified by a blunting, vertical compaction or inverted pattern of the primary biomass structure due to loss of top-predators or alteration of primary producers, with potential consequences for ecosystem functions, such as habitat provisioning and community control mechanisms [43–45]. How various environmental and biological circumstances influence the shape of biomass pyramids or the biomass size spectra slope has been successfully demonstrated in limnetic [44] and marine pelagic systems (see [46]), but we are unaware of examples from benthic marine ecosystems. Several previous studies from the Swedish west coast have described in detail the temporal (2–3 years) and spatial (2–16 meadows) variation (e.g. [18, 47–50]) of *Zostera* and the associated fauna but there are no previous attempts to characterize the eelgrass food web composition and biomass distribution among trophic levels and how it differs between environmental settings.

In this work, seagrass scientists of different specialties carried out a holistic snap-shot investigation with the aim of providing a detailed characteristic of eelgrass meadow and food web structure in two contrasting environmental settings: a wind exposed bay with relatively low nutrient levels, and a neighboring more eutrophicated, sheltered inlet with limited connection to the open sea. Seasonal and spatial variability within the ecosystems was beyond the scope of the study. We hypothesized that the sheltered, eutrophicated setting, relative to the exposed and less nutrient-rich setting, would (1) show a meadow structure with lower eelgrass biomass and higher biomass of opportunistic algae, (2) support lower diversity of consumers, (3) be characterized by a simplified food web with lower trophic diversity and (4) show a trophic level biomass distribution departing from an expected pyramidal shape.

## Materials and Methods

### Study area

**Fig 1:**
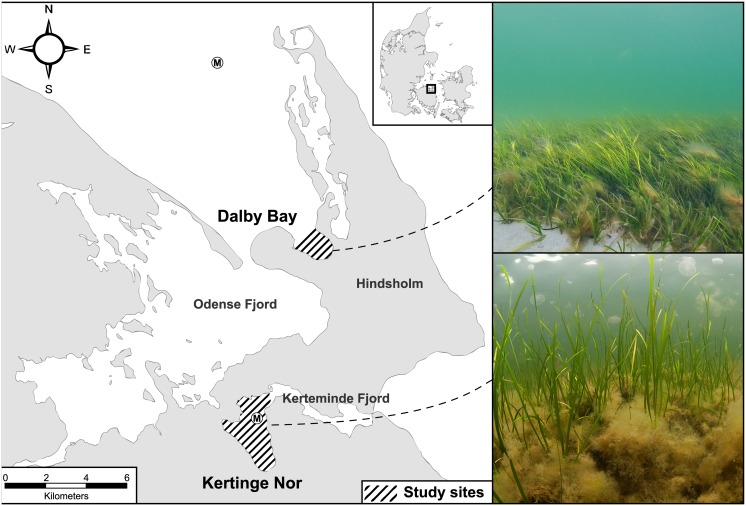
Map and photos of the study areas on Funen Island, Denmark. Hatched areas indicate the studied eelgrass meadows in Dalby Bay (55°31ʹ07ʺ N, 10°37ʹ05ʺ E) and Kertinge Nor (55°26ʹ52ʺ N, 10°33ʹ30ʺ E) while photos illustrate differences in filamentous macroalgae, eelgrass shoot density, and jellyfish abundance. Encircled M shows location of monitoring stations for water column data. Contains data from the Map 10 data set of the Danish Geodata Agency, 2015.

This study was conducted in an open (Dalby Bugt, hereafter DB, 55°31′07″ N, 10°37′05″ E) and an enclosed (Kertinge Nor, hereafter KN, 55°26′52″ N, 10°33′30″ E) bay on Funen Island, Denmark (Fig 1). The bays are located 5 km apart on opposite sides of the Hindsholm peninsula and therefore exposed to different environmental conditions. Sites were selected based on the differences in wind exposure, “openness” of the waterbodies, and history of eutrophication. Physico-chemical parameters were furthermore compiled or quantified to assess the differences between the systems as possible explanatory factors. Both areas are brackish (13–23 psu) and similar in size (2.6 and 5.4 km^2^, respectively) and in catchment area (see below).

DB is fully connected to the sea and exposed to westerly winds. DB has an average depth of 3.8 m and a maximum depth of 11 m. The sediment is dominated by bare sand with scattered patches of mainly *Fucus* spp. Eelgrass (*Z*. *marina*) grows throughout the bay from 1.5 m to 4.5 m depth [51]. Nutrient input is limited and there are no important point sources from its catchment (18.3 km^2^), except for the proximity to the entrance of Odense Fjord, where nutrients are transported to the sea [51, 52].

KN is a sheltered, shallow (2.5–3 m) embayment with a catchment area of 17 km^2^ [53]. The sediment consists of sand and mud [54] and eelgrass is widespread throughout the bay ([55], personal observation). Because KN is connected to the Great Belt through the Kerteminde Fjord which ends in a shallow and narrow channel (20–40 m wide, 750 m long), the system has limited water exchange and an average water residence time of six weeks [52, 56]. KN has a history of severe nutrient enrichment due to direct input of sewage, but after this was banned in the late 1980s annual land-based discharges of nitrogen and phosphorous were reduced by 43% and 92%, respectively [52]. Currently, the nutrient load is mainly from agriculture and linked to freshwater run-off [52]. In spite of the marked reductions in nutrient load KN is still relatively nutrient-rich and therefore referred to as “eutrophicated”.

The biological structure in KN has been the focus of previous investigations describing it as an inherently unstable eutrophic system dominated by eelgrass, filamentous macroalgae, three-spined sticklebacks (*Gasterosteus aculeatus*) and highly effective suspension feeders [52, 57]. Grazing on phytoplankton by ascidians and bivalves is high in the lower part of the water column [58] while abundant jellyfish (*Aurelia aurita*) results in a zooplankton half-life of less than one day from May to September [59].

Sampling at both sites was performed on June 21–27, 2011. Samples were collected by free diving and scuba diving at depths of 1.5–2 m. The water temperature varied between 15 and 17°C during the sampling. Permission for scientific sampling was provided by the Danish Ministry for Food, Agriculture and Fishery (journal no. 2009-02530-23088) and the work did not require specific approval from the Danish Animal Experiments Inspectorate since no experimental procedures were carried out on live animals, and fish were euthanized by a method covered by the Danish regulation on the use of animals in experiments and by Annex IV of the "Directive 2010/63/EU of the European parliament and of the council of 22 September 2010 on the protection of animals used for scientific purposes”. The study did not involve endangered or protected species.

### Data acquisition

#### Physicochemistry

Physico-chemical data from 2011 were extracted from the Danish National Monitoring Database for monitoring stations adjacent to the two study sites (Fig 1) with sampling intervals of one to three weeks. Winter concentration of nutrients is outside growth season and indicative of the nutrient status in the area. We therefore calculate the yearly temporal means for concentrations of: nitrite and nitrate (NO_x_), ammonium (NH_3_-N), total nitrogen (TN), and total phosphorous (TP). Temporal mean values for the main growth season (April-September) were calculated for chlorophyll *a* (Chl*a*) and the diffuse light attenuation coefficient *K*_d_. Cores (5 cm id, n = 4) for analysis of sediment and porewater characteristics were haphazardly taken in an area of 400 m^2^ with dense eelgrass vegetation at each site, hereafter “sampling area”.

#### Meadow structure and primary producers

Eelgrass shoot density and biomass (including associated macroalgae) were measured by harvesting the total biomass within circular frames (25 cm id, n = 5) taken haphazardly in the sampling area. The leaf growth rate was measured by marking 5–8 shoots in three replicate plots within meadows at the two sites using the leaf marking technique as described in Short and Duarte [60]. Marked shoots were collected after 6–7 days. Phytoplankton for stable isotope analysis was sampled from KN using a plankton net of 20 μm mesh size (for methods, see [61]), although missing from DB.

#### Consumers

Sediment infauna (n = 6) was sampled using sediment cores (4.7 and 10 cm in diameter in DB and KN, respectively) which were pushed 10 cm into the sediment and sealed with rubber stoppers. Mobile and sessile eelgrass epifauna (DB: n = 7; KN: n = 6) were collected using a 200 μm mesh bag on a frame enclosing an area of 35 x 35 cm [48]. Predatory fish of intermediate size were collected during day-time using a Norwegian beach seine that mainly catches fish in the size range 4–14 cm. The beach seine was 40 m long, 3.7 m high and had a mesh size of 10 mm (stretched mesh size 15 mm) in the arms and 5 mm in the central section and was towed by 20 m ropes. The seine was launched in a half circle using the shoreward seagrass edge as a baseline (for method, see [18, 62]). One haul covering approximately 250 m^2^ was taken at each site. A haul of this very large areal extension is appropriate to get less abundant species and describes the fish fauna well [18, 62]. During beach seining jellyfish *(A*. *aurita)* as well as three-spined stickleback had mass occurrences in KN, but were rare in DB. To examine possible food competition on zooplankton between the three-spined stickleback [63] and jellyfish in KN [59], we sampled jellyfish and zooplankton for SI analysis [64]. Jellyfish were sampled from the beach seine and zooplankton was collected by towing a 63 μm plankton net 20 times from the water surface to the bottom of the seagrass bed.

### Sample processing and analysis

#### Sediment, meadow structure and primary producers

The sediment cores were sliced down to 10 cm in 8 sections of 1–2 cm. The wet density of each slice was determined by weighing a known volume of sediment. Porewater was extracted from a subsample of the wet sediment by centrifugation (5–10 min at 1500 rpm) and analyzed for sulphide and ammonium [65, 66]. The remaining wet sediment was dried at 105°C for min. 12 hours to determine dry weight for calculation of water content. A homogenized subsample of the dry upper sediment layer was analyzed for particulate organic carbon and nitrogen and δ^13^C and δ^15^N isotopes (as described below) for evaluation of the eelgrass contribution to sediment carbon burial and the food source contribution of detritus to the fauna. To estimate sediment organic content, the remaining dry sediment was combusted at 520°C for 5 hours and weighed to calculate weight loss. Average values of sediment wet density, water content and organic matter content, and of porewater sulphide and ammonium were calculated for each core taking into account the varying thickness of the sections.

The maximum length of all eelgrass shoots was measured and the number of shoots was counted to estimate shoot density. Canopy height was calculated from average maximum length of shoots in each frame, and weighted by number of shoots. The samples were rinsed in freshwater, separated into leaves, rhizomes + roots and macroalgae and dried to constant weight for 24h at 60°C. Epiphytic cover of eelgrass leaves was negligible and therefore not considered separately. Leaf area index (LAI) was estimated from the ratio between leaf surface area and shoot weight measured on subsamples (n = 20) multiplied by the total leaf biomass per ground area. The aboveground production of eelgrass shoots was measured from the length of new tissue produced during the marking period and the weight to length ratio of the youngest mature leaf (leaf # 3) [60] while aboveground production on an area basis was estimated as the product of shoot production rate and shoot density.

#### Consumers

The infaunal core samples were sieved through a 500 μm mesh. Remains on a 250 μm sieve was initially checked and found almost empty which is why the 500 μm sieve was chosen. All fauna were counted under a preparation microscope and identified to the lowest taxonomic level possible, usually species or genus level. The dry weight (DW) of each species/taxon was recorded and converted to ash free dry weights (AFDW) using conversion factors for benthic fauna [48, 67]. Epifauna were removed from the leaves and sieved through a 1000 μm and a 250 μm sieve. All fauna was identified and counted. Animals retained on the 250 μm sieve were converted from abundance to AFDW using conversion factors developed in [48]. For the fauna retained on the 1000 μm sieve we used a conversion from wet weight (WW) to AFDW [67–69]. Identification of infauna and epifauna was done following the taxonomic nomenclatures in the World Register of Marine Species [70].

Fish from both sites were identified to species level, counted, measured for length, weighed and subsequently released. Due to numerous sticklebacks in KN only a subsample of the total stickleback volume was quantified. Fish for stomach content and stable isotope analysis were euthanized by percussive blow to the head followed by freezing. Because density and biomass per unit area (m^2^) of intermediate sized predatory fish are underestimated using the beach seining method a conversion factor of 3.5 (after thorough correlation with drop trap samples) was used [18, 47]. A WW to DW relationship was calculated from drying 10 three-spined sticklebacks and 8 black gobies (*Gobius niger*) at 60°C for 24 hours. The stickleback value was used as representative for sticklebacks (*G*. *aculeatus*, *Pungitius pungitius*, *Spinachia spinachia*), pipefishes (*Syngnathus rostellatus*, *Syngnathus typhle*, *Nerophis ophidion*) and garfish (*Belone belone*). The gobid value was used as representative for gobies (*G*. *niger*, *Gobiusculus flavescens*, *Pomatoschistus minutus*), rock gunnel (*Pholis gunnellus*), sculpin (*Taurulus bubalis*) and eelpout (*Zoarces viviparus*). AFWD for each species could then be calculated using the DW to AFDW conversion factor of 0.861 (Baden et al., unpublished data). Stomach content of the dominant fish species in KN (stomach samples, n = 52) and in DB (stomach samples, n = 48) were analyzed using stereomicroscopes (S3 Table).

#### Food web properties

Stable isotope analysis of δ^13^C and δ^15^N has proved valuable in food web studies where the aim is to investigate trophic levels, energy flows and pathways [71–73]. Hence, stable isotope (SI) signatures of carbon (δ^13^C) and nitrogen (δ^15^N) were measured for macrofauna, including fish and jellyfish, zooplankton, phytoplankton, benthic primary producers, and the detrital component of the sediment. In fish, shrimps, crabs and large bivalves we used muscle tissue in the analyses since this tissue has been shown to reflect long-term absorption of carbon and nitrogen [74]. In addition, invertebrates (except jellyfish and zooplankton) were kept alive overnight, allowing them to clear their guts before δ^13^C and δ^15^N analyses. This was especially important for smaller invertebrates where the entire organism was dried, grounded and used in the SI analysis. Very small or less abundant invertebrate species sometimes had to be pooled or grouped into family to obtain enough biomass for SI analysis. Phytoplankton and zooplankton samples were handled as in Jaschinski et al. [61], in brief filtered through 63μm Whatmann GF/F filters and dried to constant weight, and then analyzed for δ^13^C and δ^15^N. For eelgrass, fresh leaves, decaying leaves, roots, and rhizomes were analyzed separately.

To investigate the trophic structure the trophic position of each species was combined with its biomass for allocation to discrete trophic levels (TL) as defined by the number of steps in a linear food chain. Level 1 contains benthic primary producers, and only the biomass of macroalgae was allocated to this level. Based on the stable isotope signal combined with the very low biomass of species (e.g. Rissoids) showing an eelgrass signal we regarded the contribution from eelgrass as a food source to be negligible. Consumers often feed on several trophic levels, and the biomass of each species was assigned either to a single trophic level or divided on multiple levels depending on their food sources determined from the δ^15^N signature, SIAR mixing models, stomach analysis (when performed), and available literature (S4 Table). Thus, if the mixing model estimated a consumer to obtain 50% of its biomass from primary consumers, and 50% from secondary consumers, then half of its biomass was assigned to TL3, and half to TL4. This procedure was practiced in Jephson et al. [49] and a similar approach suggested by Trebilco et al. [46].

A way to investigate differences in trophic structure is to look at the overall slope of the regression of biomass against each TL [44] or within a range of TL [46]. A negative slope (decrease in biomass with TL), for example, forms a pyramidal structure and indicates stability in complex food webs [75, 76]. Here we estimated the relationship between biomass and trophic level for each site by plotting trophic level (TL) against log_10_ AFDW biomass pr. unit area (m^2^) and calculating the slope.

### Data analysis

Biodiversity of fauna was examined using Shannon’s diversity index (H′, bits) on abundance data. Differences in sediment, plant and faunal parameters between sites were determined through t-tests. If not stated differently all mean values are presented as mean ± SE. Sulphide data was log_10_ transformed to meet assumptions of homoscedasticity for the equal variances t-test. The slope of the overall change in log_10_(biomass) with trophic level (TL) was calculated by simple linear regression. The δ^13^C and δ^15^N values of primary sources were statistically tested amongst sources, using a one-way ANOVA, provided homogeneity of variance as tested by Bartlett’s test. Tukey’s post hoc tests were used for pairwise comparisons.

Layman et al. [77] introduced the convex hull, describing the area of the isotopical (δ^13^C and δ^15^N) niche and representing a quantitative indicator of nutritional niche space and therefore trophic diversity. The total area (TA) of the convex hull was calculated from the area covered by the whole food web as indicated by the species’ average values in the δ^13^C—δ^15^N space.

The mass-balance model IsoSource 1.3 [78] with increments of 1% and tolerance of 0.1, was used to evaluate the contribution of primary producers to sediment organic matter, based on their δ^13^C signatures. The Bayesian mixing model SIAR version 4.1 (Stable Isotope analysis in R [79]) was used to assess the relative contribution (%) of food sources to the consumers. SIAR was selected as it allows the incorporation of source variation into the analysis. Selection of a potential food source to a consumer was based on the abundance of the food source in each location, prior knowledge on the diet of the consumer, and for the larger consumers, stomach contents. A value of 1.5±0.5 δ^15^N was used as trophic enrichment for the primary consumers and a value of 3±0.5 δ^15^N for consumer discrimination following Jaschinski et al. [61]. We choose an average discrimination factor of 0.5±0.2 δ^13^C for estuarine systems [80]. All statistical analyses were conducted using R 3.03 [81].

## Results

### Physicochemistry

Water column nitrogen concentrations were higher in KN than in DB, with nitrite and nitrate concentrations six times as high (6.6 vs. 1.1 μmol l^-1^), ammonium concentration four times as high (2.1 vs 0.5 μmol l^-1^) and total nitrogen concentration twice as high (27.9 vs. 14.2 μmol l^-1^) (Table 1). Total phosphorous concentration was about 2.5 times as high in DB (2.24 μmol l^-1^) as in KN (0.87 μmol l^-1^), although the high value in DB was caused by a single spike in April following a phytoplankton bloom in March. Similarly high TP concentrations have not been recorded in DB in the past 25 years, and when excluding this spike, DB has a TP concentration of 0.65 μmol l^-1^ in 2011 which is 25% lower than in KN. Chlorophyll *a* concentration was 2.3 times as high in KN (5.21 μg l^-1^) as DB (2.24 μg l^-1^) while the diffuse light attenuation *K*_d_ was twice as high in KN (0.68) compared to DB (0.30) indicating less light reaching the eelgrass in KN. Seasonal mean and max temperature is higher in KN (14.6°C and 21.4°C) than in DB (13.3°C and 17.7°C) which reflects faster warming and restricted water exchange in the shallow inlet KN.

**Table 1:** Monitoring data for water column concentrations of nutrients, chlorophyll a, light attenuation and temperature.

Monitoring variable	DB			KN		
	mean	(min—max)	n	mean	(min—max)	N
N0_x_ (μmol l^-1^)	1.10	(0.11–7.86)	20	6.62	(0.11–25.71)	32
NH3-N (μmol l^-1^)	0.53	(0.21–2.36)	20	2.06	(0.21–10.00)	32
TN (μmol l^-1^)	14.20	(10.71–62.14)	20	27.93	(16.43–41.43)	32
TP (μmol l^-1^)	2.14	(0.29–25.16)	20	0.87	(0.55–1.84)	32
Chl*a* (μg l^-1^)	2.24	(1.23–8.60)	13	5.21	(1.56–13.20)	18
*K*_d_ (coefficient)	0.30	(0.21–0.41)	13	0.68	(0.41–1.30)	18
Temperature (°C)	13.26	(2.35–17.70)	13	14.63	(5.81–21.44)	20

Temporal mean values calculated from sampling intervals of 1–3 weeks in DB and KN, 2011. Number of samplings indicated by n. Yearly means and range are given for nitrogen and phosphorous concentrations, and main growing season (April-September) means and range for chlorophyll a, the diffuse light attenuation coefficient (*K*_d_) and temperature.

Sediment wet density and water content were similar in DB and KN while organic content in KN was 2.3 times as high compared to DB (Table 2). Porewater concentrations of dissolved sulphide (range 0–14.6 μM) were also significantly higher in KN compared to DB, but values were low at both sites. Similarly, porewater concentrations of dissolved ammonium (range 11.1–65.6 μM) were low but showed no significant difference between sites. C:N ratios of the top 1 cm sediment layer were twice as high in DB compared to KN, reflecting a significantly higher nitrogen content in KN. The stable isotope analysis revealed a sediment δ^13^C of -18.8‰ in DB and -18.1‰ in KN. Sediment δ^15^N was 6.0 and 4.9‰, respectively (Table 3). The contribution of primary sources to the sediment pools from eelgrass (leaves, rhizomes and roots) was low at both sites (DB: 0–8 and KN: 0–9%), potentially higher from macroalgae (DB: 0–45 and KN: 0–49%) and highest from phytoplankton (DB: 55–92 and KN: 51–92%).

**Table 2:** Physical and biogeochemical sediment parameters (mean ± SEM) from DB and KN (n = 3–4).

Parameter	DB	KN	t-test
Wet density (g cm^-3^)	2.29±0.02	2.34±0.03	p = 0.299
Water content (% of WW)	18.9±0.5	21.4±0.3	p = 0.006
Organic matter (% of DW)	0.30±0.03	0.70±0.02	p < 0.001
Sulphide (μM)	0.27±0.07	6.30±2.46	p = 0.010
Ammonium (μM)	28.2±2.5	55.0±20.8	p = 0.249
C:N ratio^1^	33.0±5.6	17.0±2.4	p = 0.032
POC (% of DW)	0.64±0.02	0.63±0.01	p = 0.709

Values represent the top 10 cm of sediment if not otherwise stated.

^1^Of top 1 cm sediment. C:N calculated on molar basis.

**Table 3:** Stable isotope δ^13^C and δ^15^N values (mean ± SEM) of primary sources and animal species associated to seagrass meadows in and Dalby Bay and Kertinge Nor in Denmark, June 2011.

			Dalby Bay		Kertinge Nor		
Taxa	n	Ti	δ^13^C	δ^15^N	δ^13^C	δ^15^N	Ab.
**SEDIMENTS**							
Detritus	4	S	-18.78±1.07	6.00±0.67	-18.11±0.65	4.91±0.2	Det
**PRIMARY PRODUCERS**							
Phytoplankton (bulk)	3	W	-	-	-19.06±0.12	6.58±0.21	PP
**Chlorophyta**							
*Chaetomorpha* sp.	3	W	-	-	-15.69±0.45	8.84±0.61	Ma
*Cladophora* sp.	1	W	-	-	-17.68	5.16	Ma
*Ulva lactuca*	2	W	-	-	-16.55±0.56	7.57±0.22	Ma
*Ulva intestinalis*	1	W	-11.39	7.07	-	-	Ma
**Phaeophyta**							
*Ectocarpus* sp.	1/2	W	-19.17	5.38	-19.09±0.18	6.4±0.03	Ma
**Rhodophyta**							
*Ceramium tenuicorne*	1	W	-	-	-16.89	8.50	Ma
*Gracilaria* sp.	1	W	-16.73	7.37	-	-	Ma
*Polysiphonia* sp.	1	W	-20.63	6.03	-	-	Ma
***Zostera marina***							
Fresh leaves	3	W	-6.56±0.68	7.39±1.16	-7.34±0.14	6.67±0.37	Zm
Decaying leaves	3	W	-	-	-8.25±0.04	6.89±0.16	
Roots	3	W	-6.73±0.31	6.93±1.33	-7.39±0.32	6.67±0.51	Zm
Rhizomes	3	W	-6.94±1.01	5.94±1.65	-7.14±0.65	5.73±0.43	Zm
**INVERTEBRATES**							
**Bivalvia**							
*Mytilus edulis*	3	M	-	-	-17.05±0.1	8.85±0.5	Myed
*Mya arenaria*	1	W	-	-	-13.13	7.53	Myar
**Cnidaria**							
*Aurelia aurita*	6	P	-	-	-20.25±0.59	12.3±0.24	Auau
*Haliclystus auricula*	1	W	-20.64	10.47	-	-	Haau
**Crustacea**							
Amphipoda spp. (< 2 mm)	4	P	-22.21±0.59	7.54±0.13	-	-	Amp
*Carcinus maenas*	3	M	-17.39±0.75	11.86±0.4	-14.41±0.27	11.97±0.24	Cama
*Crangon crangon*	3	M	-13.75±0.18	13.16±0.17	-	-	Crcr
*Gammarus locusta*	2/5	P	-21.00±0.25	7.90±0.39	-18.61±1.04	7.52±0.34	Galo
*Idotea* spp.	3	P	-17.04±0.35	8.28±0.05	-14.39±0.84	6.91±0.47	Ido
*Microdeutopus gryllotalpa*	3	P	-	-	-17.65±0.52	5.30±0.14	Migr
*Monocorophium insidiosum*	3	P	-	-	-16.41±0.20	6.89±0.26	Moin
*Palaemon adspersus*	3	M	-15.06±0.23	11.60±0.33	-13.62±0.05	11.49±0.38	Paad
*Palaemon elegans*	3	M	-16.96±0.41	11.98±0.25	-13.76±1.23	11.29±0.03	Pael
Zooplankton (bulk)	1	P	-	-	-19.54	7.49	ZP
**Echinodermata**							
*Asterias rubens*	3	W	-15.10±0.19	10.81±0.43	-14.14±0.5	10.1±0.19	Asru
**Gastropoda**							
*Littorina* sp.	1	P	-	-	-11.94	5.75	Lisp
*Littorina littorea*	3	P	-16.50±0.5	8.89±0.73	-	-	Lili
*Littorina obtusata*	3	P	-12.27±3.19	9.08±0.1	-	-	Liob
*Littorina saxatilis*	1	P	-	-	-13.79	6.38	Lisa
*Hydrobia* spp.	1	P	-11.50	7.35	-	-	Hyd
*Rissoa* spp. & *Hydrobia* spp.	7	P	-	-	-8.54±0.32	6.77±0.13	RiHy
**Polychaeta**							
*Nereidinae* spp.	1	P	-18.18	10.38	-16.29	10.54	Ner
**VERTEBRATES**							
**Ascidiacea**							
*Ciona intestinalis*	3	W	-	-	-16.69±0.22	7.28±0.13	Ciin
**Fish**							
*Belone belone*	1	W	-	-	-15.38	13.07	Bebe
*Gasterosteus aculeatus*	3	M	-21.91±0.46	14.69±0.33	-14.39±0.66	13.39±0.14	Gaac
*Gobius niger*	3/6	M	-17.53±1.45	12.53±1.06	-13.99±0.37	12.73±0.10	Goni
*Nerophis ophidion*	3	M	-18.56±0.49	12.15±0.33	-	-	Neop
*Pholis gunnellus*	3	M	-17.45±0.35	13.62±0.43	-	-	Phgu
*Pomatoschistus minutus*	3	M	-16.67±0.44	13.11±0.54	-	-	Pomi
*Pungitius pungitius*	3	M	-	-	-15.45±0.45	12.46±0.53	Pupu
*Spinachia spinachia*	3	M	-19.51±0.16	10.52±0.06	-	-	Spsp
*Syngnathus rostellatus*	3	M	-21.96±0.67	12.57±0.26	-	-	Syro
*Syngnathus typhle*	3	M	-19.46±0.61	14.70±0.98	-16.18±0.77	12.44±0.35	Syty
*Taurulus bubalis*	3	M	-16.41±0.22	14.70±0.32	-	-	Tabu
*Zoarces viviparus*	3	M	-17.71±0.57	12.94±0.15	-13.97±0.11	12.52±0.08	Zovi

Ti = Tissue; M = muscle tissue; P = pooled whole organisms; S = top sediment layer; W = whole organism; dash (-) = not present or not sampled; Ab. = abbreviation. Identical abbreviations indicates sample values that have been pooled within site on Fig 5.

### Meadow structure and primary producers

**Fig 2:**
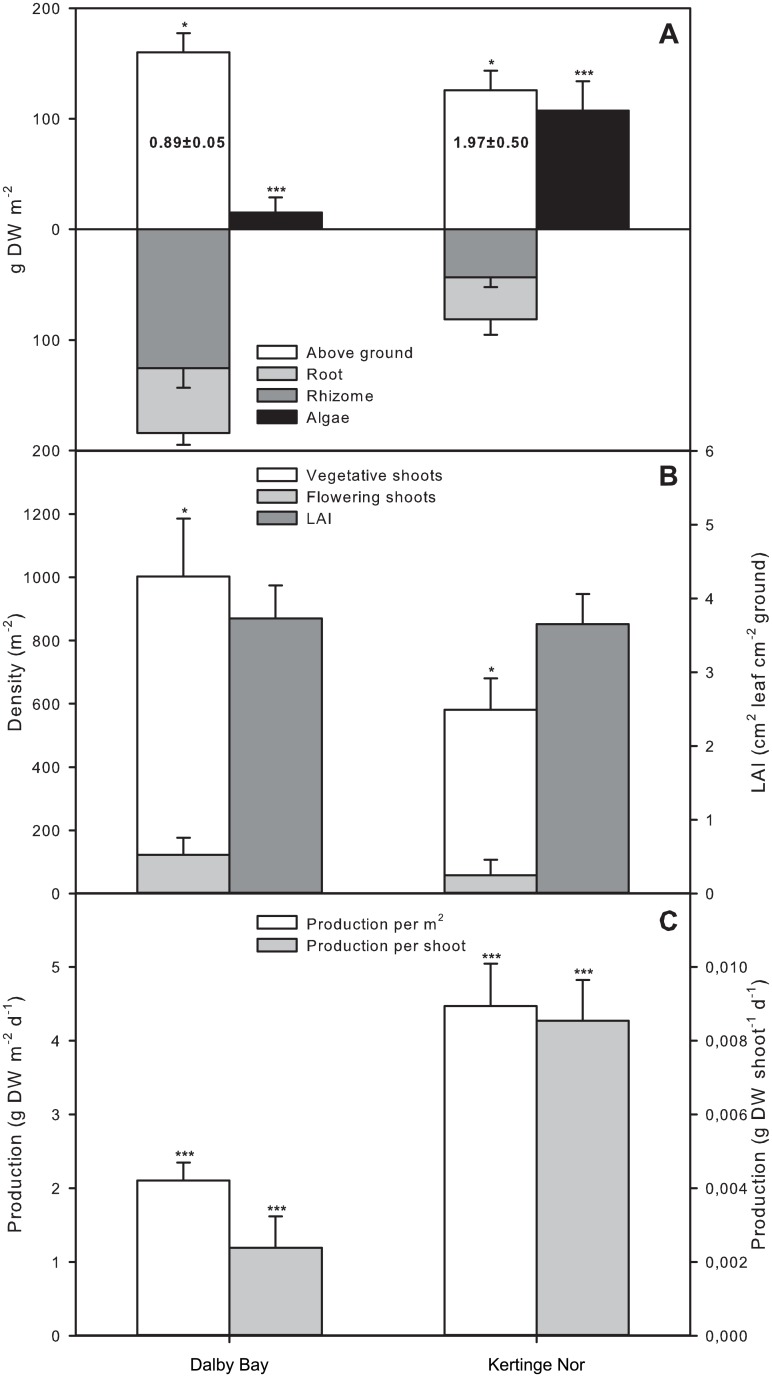
*Zostera marina* meadow characteristics in Dalby Bay and Kertinge Nor. Values are mean ± SE. Asterisks indicate statistical significant differences based on t-tests (* = 0.05, *** = 0.001). For stacked columns the statistics refer to the summed value. (A) *Z*. *marina* and algal biomass. Numbers in columns are above to below ground biomass ratios. (B) *Z*. *marina* shoot density and leaf area index. (C) *Z*. *marina* above ground production.

Eelgrass biomass (t-test, p = 0.044, n = 5) and shoot density (t-test, p = 0.044, n = 5) were significantly higher in DB compared to KN (Fig 2A and 2B) while the above- to below-ground biomass ratio was higher in KN (KN: 1.97±1.12 *vs*. DB: 0.89±0.11, t-test, p = 0.021, n = 5). The leaf area index (LAI) was similar at the two sites (Fig 2B) as the lower shoot density at KN was compensated by taller canopies (KN: 41.9±1.5 cm vs. DB: 22.3±0.6 cm, t-test, p = 0.001, n = 5). Likewise we found no difference in the proportion of flowering shoots (DB: 11.6±3.7, KN 8.6±6.8%, t-test, p = 0.703, n = 5, Fig 2B) or mean shoot biomass (DB: 0.39±0.07 vs. KN 0.37±0.04 g DW shoot^-1^, t-test, p = 0.813, n = 5) between the two sites.

However, leaf elongation rate was significantly higher in KN compared to DB (DB: 18.4±1.87 mm d^-1^ vs. KN 53.0±6.22 mm d^-1^, unequal variances t-test, p < 0.0001, n = 20), and so was eelgrass above-ground production when calculated per shoot (t-test, p<0.0001, n = 20) as well as per unit area (t-test, p = 0.0003, n = 20) (Fig 2C). Macroalgal biomass was 7 times higher in KN compared to DB (t-test, p = 0.014, n = 5, Figs 1 and 2A). The macroalgae primarily consisted of drifting opportunistic species dominated by *Chaetomorpha linum*, *Cladophora* cf. *seriacea* and *Ectocarpus* sp. The combination of lower eelgrass biomass and higher biomass of opportunistic algae at KN compared to DB reflects a major difference in the dominance pattern of benthic primary producers in the two contrasting ecosystems.

### Consumers

**Fig 3:**
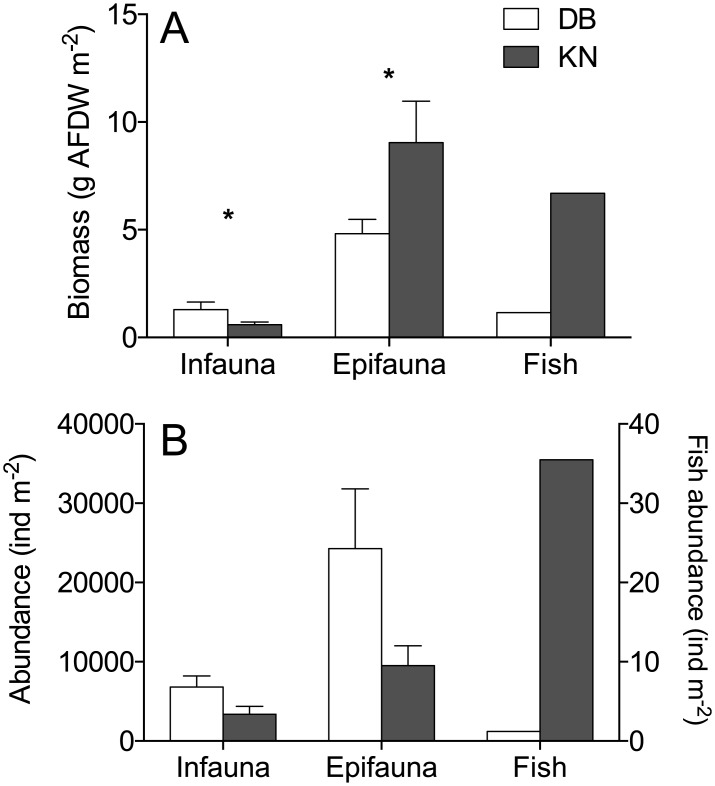
Faunal biomass (A) and abundance (B) in Dalby Bay and Kertinge Nor in June 2011. Asterisks indicate significant difference between the two sites (t-test <0.05).

**Fig 4:**
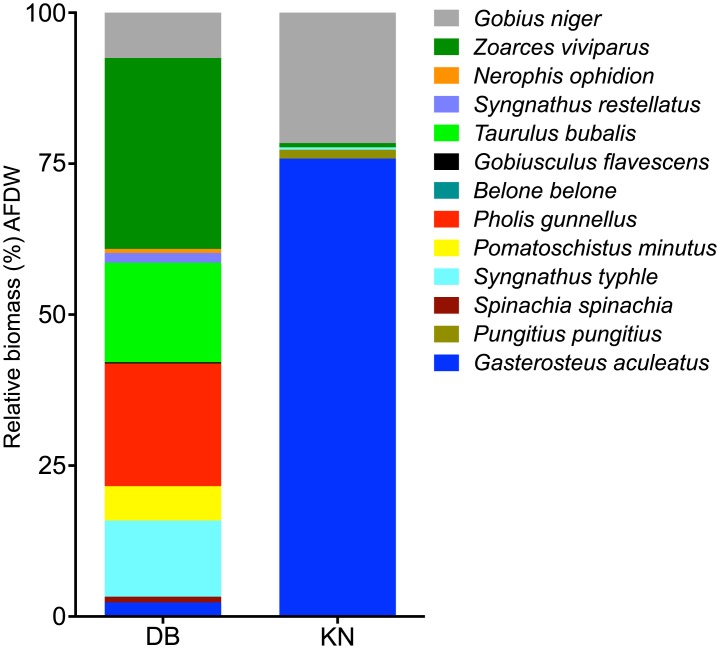
The relative (%) species contribution to total fish biomass (AFDW m^-2^) in Dalby Bay (DB) and Kertinge Nor (KN) in June 2011.

The faunal community composition and biomass at KN and DB differed in many aspects (Figs 3 and 4, S1 and S2 Tables). The total biomass of all invertebrates and fish was 16 g AFDW m^-2^ in KN and 8 g AFDW m^-2^ in DB. The contribution of infauna to the total community biomass was small at KN (4%) and moderate at DB (20%). In DB the annelids *Alitta virens* and *Hediste diversicolor* dominated the infauna, whereas Hydrobiid snails and oligochaetes dominated in KN. Shannon’s index of diversity H’ did not differ significantly between the infaunal communities of the two sites (DB: 1.63±0.099 vs. KN: 1.52±0.048, t-test, p = 0.36, n = 5).

Due to low relative biomass, the species *Pungitius pungitius* in DB (0.02%) and *Belone belone* in KN (0.004%) are not distinguishable on the figure. See S2 Table for values.

The invertebrate epifauna contributed most to the total faunal community biomass in both sites, i.e. 55% (9.0 g AFDW^-2^) in KN and 64% (4.8 g AFDW m^-2^) in DB. In KN, the shrimp *Palaemon adspersus* made up about 50% of the total epifaunal biomass followed by the amphipods *Monocorophium insidiosum* and *Microdeutopus gryllotalpa* which together constituted about 20%. In DB, the small (2-3mm) amphipod *M*. *insidiosum* constituted 40% of the total epifaunal biomass, whereas the rest of the biomass comprised Rissoid and Littorinid snails and spat of *Mytilus edulis*. The mesograzers *Gammarus locusta* and *Idotea balthica* had similar combined abundance and biomass at KN and DB, i.e. 77 ind. m^-2^ (0.4 g AFDW m^-2^) and 53 ind. m^-2^ (0.2 g AFDW m^-2^), respectively. Shannon’s index of diversity H’ did not differ significantly between the epifaunal communities of the two sites (DB: 1.23±0.059 vs. KN: 1.36±0.141, t-test, p = 0.426, n = 6–7).

The fish assemblage differed significantly between the two locations. DB supported twice (12 species) as many species as KN, but the biomass in DB was low being 1.2 g AFDW m^-2^, in contrast to 6.7 g AFDW m^-2^ in KN. This represents 42% of the consumer biomass in KN and 15% in DB but with a sample size of one, some uncertainty remains despite the large areal extension of the beach seine hauls. The fish community in DB was also dominated by larger stationary species with lower abundance than in KN. The higher diversity in DB is also reflected in the Shannon’s diversity index H´ (DB: 2.13 vs. KN: 0.40). In DB, dominant species were eelpout (*Z*. *viviparus*, mean length 12 cm), broad-nosed pipefish (*S*. *typhle*, 7–17 cm) and rock gunnel (*P*. *gunnellus*, mean length 16 cm) (Fig 4, Table 3). The fish community in DB reached a density of only 1.3 ind. m^-2^. In KN, the intermediate predatory fish assemblage was almost completely dominated by three-spined sticklebacks (*G*. *aculeatus*, mean length 4.3 cm) and black gobies (*Gobius niger*, mean length 6.1 cm) and the total fish abundance reached 36 ind. m^-2^ (Fig 3).

### Food web properties

**Fig 5:**
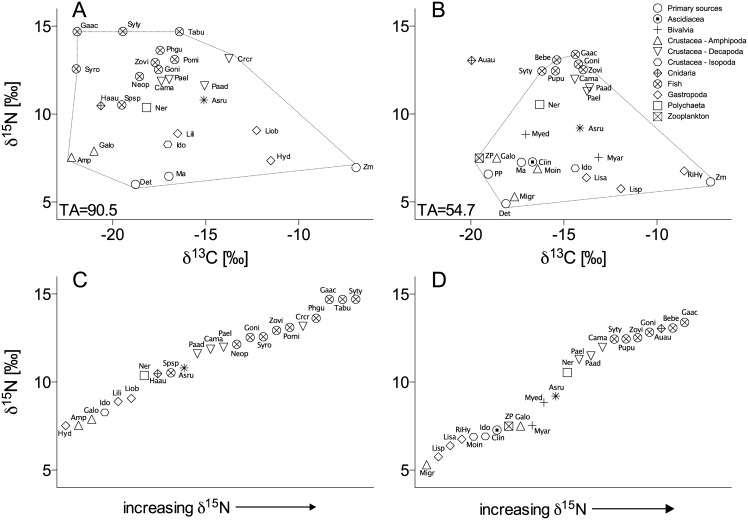
Stable isotope values of species associated to *Zostera marina* meadows in Dalby Bay (DB) and Kertinge Nor (KN) in Denmark, June 2011. Mean δ^13^C vs. δ^15^N in (A) DB and (B) KN. Ascending mean δ^15^N values of consumer species in (C) DB and (D) KN. The dashed line indicates the convex hull. TA is the total area of the convex hull. *A*. *aurita* was excluded from the TA due its special position in the food web and lack of sampling in DB; See Table 3 for species abbreviations and isotopic signals.

In DB, δ^13^C and δ^15^N of primary producers ranged from -20.0 to -6.9‰ and 5.9 to 6.8‰, respectively. In KN the corresponding numbers were -19.5 to -7.1‰ and 4.9 to 7.5‰ (Fig 5, Table 3). At both sites, eelgrass δ^13^C represented a distinct group separated from macroalgae and detritus (ANOVA, p<0.01, Tukey test), whereas the δ^15^N did not vary significantly between primary producers. The δ^13^C of consumers ranged from -22.2‰ (Amphipoda spp.) to -11.5‰ (*Hydrobia* spp.) in DB, and from -20.2‰ (*Aurelia aurita*) to -8.5‰ (*Rissoa* spp. & *Hydrobia* spp.) in KN. The δ^15^N ranged from 6.1‰ (*Macoma balthica*) to 14.7‰ (*G*. *aculeatus*, *S*. *typhle*, *T*. *bubalis*) in DB and from 5.3‰ (*M*. *gryllotalpa*) to 13.4‰ (*G*. *aculeatus*) in KN (Table 3).

The two dimensional isotopic space illustrated a 60% larger convex hull area in DB compared to KN (Fig 5A and 5B), indicating that DB had a more complex food web with higher trophic diversity and thus broader nutritional niche space. This is mainly due to a broader spatial spread of secondary consumers in the isotopic space of DB as compared with KN, where the secondary consumer values are more centralized.

In both systems, the mesograzers (mainly *Idotea balthica* and *Gammarus locusta)* were more closely associated with carbon sources originating from macroalgae and/or detritus, while the eelgrass derived carbon appeared to be more important for the gastropods *Littorina obtusata* and *Hydrobia/Rissoa* spp. [49, 82]. This is also supported by the SIAR results of primary consumers (Table 4). The consumption of eelgrass by gammarid amphipods was marginal (3%). Idoteids received more eelgrass derived carbon than the gammarids, especially in KN (KN: 28% vs. DB: 7%). The relative importance of eelgrass in the diets of hydrobiid and rissoid snails was similarly higher in KN compared to DB (KN: 86% vs. DB: 52%).

**Table 4:** Proportional (%) contribution of primary production food sources to selected benthic invertebrates in DB and KN in June 2011.

Primary consumers	Food sources		
	*Zostera marina*	Macroalgae	Detritus
**DB**			
**Crustacea:**			
*Amphipoda* spp.	4 (0–41)	40 (2–71)	43 (8–79)
*Gammarus locusta*	3 (0–42)	43 (3–73)	45 (6–79)
*Idotea* spp.	7 (0–35)	42 (5–74)	46 (10–78)
**Gastropoda:**			
*Hydrobia* spp.	52 (42–59)	17 (0–47)	27 (4–46)
*Littorina littorea*	11 (0–37)	41 (2–76)	45 (6–78)
*Littorina obtusata*	44 (22–66)	38 (1–58)	20 (0–48)
**KN**			
**Crustacea:**			
*Gammarus locusta*	3 (0–37)	38 (9–63)	49 (23–78)
*Idotea* spp.	28 (6–50)	17 (0–52)	43 (16–76)
*Microdeutopus gryllotalpa*	2 (0–33)	5 (0–59)	86 (28–99)
*Monocorophium insidiosum*	10 (0–25)	18 (0–54)	72 (34–89)
**Gastropoda:**			
*Littorina* sp.	41 (5–61)	31 (0–56)	39 (1–64)
*Rissoa* spp. & *Hydrobia* spp.	86 (75–93)	1 (0–14)	11 (0–19)

Central tendency from SIAR mixing models (using δ^13^C and δ^15^N combined) are given in mode (95% CI).

SIAR mixing models of secondary consumers (Tables 5 and 6) indicate that smaller amphipods constituted about 50% of the diet in the gammarids of KN, and 25% of idoteid diet at both sites. The *Palaemon* shrimps in KN mainly fed on polychaetes (38%) and snails (26%), with negligible input from crustaceans or primary sources. In DB, the diet of *Palaemon* shrimps consisted of crustaceans (24–28%), gastropods (18–46%) and polychaetes (21–32%). The fishes (*S*. *typhle*, *P*. *gunnellus*, *T*. *bubalis*, *Z*. *viviparus*) in DB showed a mixed diet of crustaceans (*Palaemon* shrimps; 14–41%) and polychaetes (4–27%). Small amphipods only contributed to the diet of smaller pipefish and rock gunnel (21–25%) while isopods were important to *Z*. *viviparus*. In KN, polychaetes (63–74%) and snails (15–19%) dominated the diet of sticklebacks and black gobies. The stomach analysis of sticklebacks and black gobies confirm the SIAR results (S3 Table). However, they also show a high abundance of small amphipods and more rarely gammarids of 6–8 mm in stickleback and black goby stomachs.

**Table 5:** Proportional (%) contribution of food sources to selected consumers in Dalby Bay, Denmark.

Food sources	Consumers								
	*Idotea* spp.	Nereidinae spp.	*Palaemon adspersus*	*Palaemon elegans*	*Carcinus maenas*	*Syngnathus typhle*	*Pholis gunnellus*	*Taurulus bubalis*	*Zoarces viviparus*
**Primary sources**									
Detritus	25 (5–38)	6 (0–33)							
Macroalgae	25 (4–39)	6 (0–33)	5 (0–15)	2 (0–18)	30 (15–45)				
**Crustacea:**									
Amphipoda spp.	28 (14–40)	50 (28–66)	5 (0–17)	23 (8–34)		21 (0–53)	25 (13–37)	4 (0–17)	3 (0–29)
*Idotea* spp.			19 (1–35)	5 (0–30)	9 (0–34)	2 (0–21)	1 (0–15)	1 (0–15)	32 (16–59)
*Palaemon adspersus*						2 (0–24)	3 (0–27)	14 (0–34)	26 (2–43)
*Palaemon elegans*						14 (0–31)	33 (10–56)	41 (18–70)	4 (0–26)
**Fish:**									
*Pomatoschistus minutus*					20 (0–34)				
**Gastropoda:**									
*Hydrobia spp*.	28 (20–36)	23 (12–31)	20 (9–30)	1 (0–11)	1 (0–14)				
*Littorina littorea*			17 (1–30)	12 (0–30)	8 (0–32)				
*Littorina obtusata*			9 (0–20)	5 (0–17)	2 (0–20)				
**Polychaeta:**									
Nereidinae			21 (8–34)	32 (16–48)	25 (9–44)	16 (0–30)	27 (0–48)	27 (3–47)	4 (0–32)

Central tendency from SIAR mixing models (using δ^13^C and δ^15^N combined) are given in mode (95% CI), and thus not necessarily sum to unity.

**Table 6:** Proportional (%) contribution of food sources to selected consumers in Kertinge Nor, Denmark.

Food sources	Consumers					
	*Aurelia aurita*	*Gasterosteus aculeatus*	*Gobius niger*	*Palaemon* spp.	*Idotea* spp.	*Gammarus locusta*
**Primary sources:**						
Macroalgae				2 (0–18)	2 (0–24)	23 (3–38)
Detritus				1 (0–9)	29 (5–53)	19 (1–33)
*Zostera marina*					23 (8–33)	
**Crustacea:**						
*Gammarus locusta*				2 (0–24)		
*Idotea* spp.		2 (0–25)	3 (0–31)	2 (0–23)		1 (0–14)
*Microdeutepus gryllotalpa*		1 (0–14)	1 (0–21)	1 (0–10)	25 (0–45)	18 (1–37)
*Monocorophium insidiosum*	2 (0–22)	2 (0–28)	2 (0–18)		33 (16–57)	
Zooplankton	48 (25–90)	1 (0–17)	2 (0–24)			
**Fish:**						
*Gasterosteus aculeatus*	5 (0–40)					
**Gastropoda:**						
*Rissoa* spp. & *Hydrobia* spp.	15 (2–26)	19 (6–32)	26 (16–33)			
**Polychaeta:**						
Nereidinae	5 (0–58)	74 (21–85)	63 (8–74)	38 (23–48)		

Central tendency from SIAR mixing models (using δ^13^C and δ^15^N combined) are given in mode (95% CI), and thus not necessarily sum to unity.

The trophic structure (Fig 5C and 5D) indicated differences in the δ^15^N baseline level of primary consumers between sites of around 2.5‰. The δ^15^N signal of larger gammarid amphipods in KN is enriched, indicating that they are both primary consumers and predators. In KN, a vertical zonation of consumers is visible (Fig 5D), roughly dividing the community in two parts; the primary consumers (δ^15^N: 5.3–7‰) and the secondary and tertiary consumers (δ^15^N: 11.3–13.4‰) with some species (large gammarids, *A*. *rubens* and *M*. *edulis*) in the transition zone between δ^15^N 7.3 and 10.5‰. In contrast, the vertical division in DB is less prominent, but a taxonomically heterogeneous group consisting of *A*. *rubens*, *Spinachia spinachia*, *Nereis diversicolor* and *Haliclystus auricula* can be discerned between δ^15^N 10–11‰ (Fig 5C).

**Fig 6:**
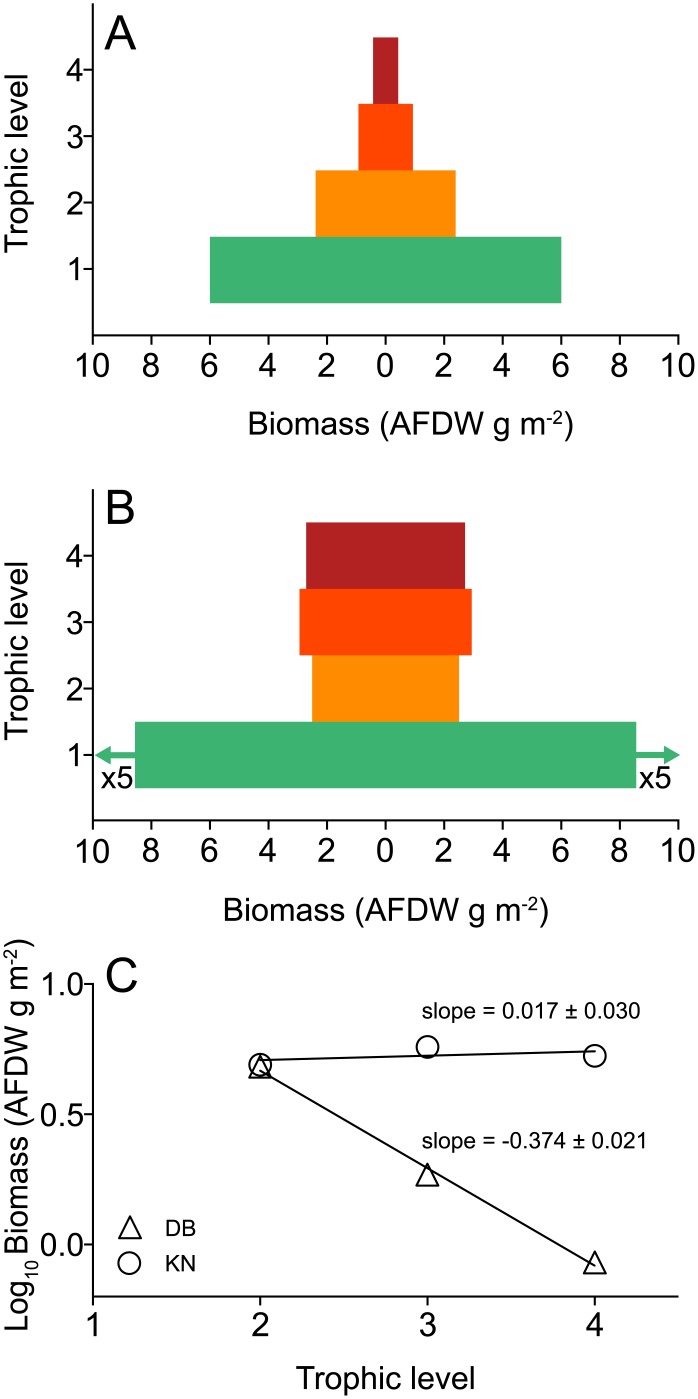
Biomass distribution at trophic levels of *Zostera marina* meadows in Dalby Bay and Kertinge Nor, Denmark, June 2011. The biomass of each species is distributed across trophic levels (TL) 1–4 according to their diet, based on stable isotope mixing model results (Tables 5 and 6), gut contents (S3 Table), and literature data (S4 Table). Combined width of bars indicate biomass at each TL in (A) Dalby Bay and (B) Kertinge Nor. Note biomass value of TL1 in KN is divided by 5 for visual purposes. (C) Linear regression of TL 2–4 and log_10_biomass (DB: log_10_biomass = 1.416–0.374×TL, R^2^ = 0.99; KN: log_10_biomass = 0.683 + 0.017×TL, R^2^ = 0.24), with slope indicating overall shape of the consumer part of biomass pyramids in Fig 6A and 6B above.

There was a clear difference in the distribution of biomass between trophic levels at the two sites. In DB, less biomass was present at the higher trophic levels as indicated by the triangular shape of the food web pyramid (Fig 6A). In KN, the consumer part (TL2-4) of the pyramid is columnar on a broad base of primary producers (Fig 6B) due to relatively more biomass at higher trophic levels. Consequently, the slope of the consumer biomass pyramids differed markedly between sites being negative (-0.374) for DB and close to zero (0.017) for KN (Fig 6C). This represents an average factor 2.4 decrease in biomass with each TL in DB, and no difference between consumer TLs in KN.

## Discussion

Our initial hypotheses were supported and the study showed that the sheltered, eutrophicated site exhibited (1) markedly lower eelgrass biomass and shoot density and larger dominance of opportunistic algae, (2) lower species diversity of fish, (3) a food web represented by high biomass of a few dominant consumer species, indicative of an ecosystem with few but strong food web links [33, 83] and (4) a column-shaped distribution of consumer biomass. The combined information on food web structure, eelgrass meadow structure and environmental setting offers detailed insight in the complex control mechanisms operating in these shallow coastal ecosystems. Such information is relevant for evaluating the susceptibility of the ecosystems to further anthropogenic pressure. Specifically, the high abundance of opportunistic algae and a simple food web structure found at the protected, eutrophicated site (Fig 5A and 5B), signals a disturbed and potentially unstable system, with eroded resilience. These features are evidenced in KN by several previous episodes of anoxia, nutrient release and subsequent intense algal blooms enhanced by abundant jellyfish controlling zooplankton (see [52, 57]). The likelihood of anoxia is larger in KN due to the combination of more organic material, slower water renewal and warmer water increasing the metabolic rates. The dense algal mats here further increase the risk [52, 84], which would be detrimental to the sediment infauna and epifauna, and thus higher trophic levels. Anoxia was indeed a common phenomenon in KN in the past when nutrient loading was higher, but such conditions were not recorded at the time of sampling, and thus haven’t affected the observed pattern of trophic level biomass distribution.

### Primary producer and sediment conditions

Reduced shoot density is a well-known response of eelgrass to shading (e.g. [85]), which in KN can be attributed to the high abundance of filamentous macroalgae and reduced water clarity (Table 1). In addition, shoot density may also be reduced by nutrient loading [85] or reduced sediments [86]. These factors are often intercorrelated, however, as water column nutrients tend to increase shading and also contribute to anoxic sediments. Low water transparency in KN together with limited wave action may also explain the taller canopy and higher above to below ground biomass ratio [87]. Despite lower eelgrass density and biomass, the above-ground eelgrass production was higher in KN compared to DB, suggesting a faster turnover of the above-ground eelgrass biomass. The opportunistic algae at KN are characterized by fast growth and biomass turnover, which contribute to faster recycling of carbon and nutrients [88] in KN compared to DB. The organic matter content at the more nutrient-rich KN was twice as high as in DB, but both were low compared to the global mean of seagrass meadows (4.1% LOI, [89]), which supports the notion of high turnover of the organic matter in these meadows. Similarly, both nutrient and sulphide pools were in the low range compared to other eutrophic habitats [90]. However, the lack of seasonal and annual replicates makes it difficult to draw further conclusions since most pore water pools fluctuate within and between years [91].

The majority of stable isotope studies show that food webs in eelgrass meadows are characterized by a large degree of omnivory and fueled mainly through algal and detrital pathways [61, 92, 93]. Correspondingly we found that for both sites phytoplankton contributed most (51–92%) to the sediment organic pool followed by macroalgae (0–45%), whereas the contribution of eelgrass (leaves, rhizomes and roots) was low (<9%). Furthermore there was a larger span between seagrass and sediment δ^13^C signatures (10.8‰ in KN and 12.2‰ in DB) compared to seagrass meadows globally (6.3‰, [89]), emphasizing that phytoplankton and filamentous algae play relatively larger roles in nutrient recycling in the Danish meadows studied here compared to the global average. Based on the δ^15^N values, both study sites showed signs of nutrient enrichment compared to pristine conditions [94]. However, the eelgrass range of δ^15^N (6.4–6.8‰) is low to intermediate compared to more eutrophic areas.

### Food sources and consumer interactions

Danish eelgrass fauna has not been thoroughly described in the scientific literature since the investigations of Blegvad in 1919 [95] and Muus in 1967 [96]. The faunal composition of eelgrass meadows at KN and DB show high similarity with eelgrass communities in Skagerrak and the southern Baltic Sea [18, 47, 50, 97]. Seasonal variation of eelgrass epifauna and fish has been thoroughly described for Swedish eelgrass ecosystems [18, 47–49]. In these seagrass beds, the species composition remain constant, despite natural interannual changes in recruitment of invertebrates and fish, suggesting that our sampling approach was sufficient to capture the majority of the eelgrass species pool. Differences in temperature potentially affect timing of faunal recruitment and subsequent growth. However, as sampling took place in June, months after the dominant phytoplankton and zooplankton spring blooms, and prior to fish recruitment [47], it is unlikely that temperature effects on recruitment played any important part in explaining the difference in trophic level biomass distribution (slope) between the sites (see below). It is also likely, that the shape of the biomass distribution will persist as e.g. the fish biomass will remain high or increase towards the end of the season [18, 47, 48]. The relative occurrence of dominant invertebrate species appeared to be related to exposure, e.g. more grass shrimps (*Palaemon adspersus*) at the sheltered site KN and more *Monocorophium insidiosum* in the relatively exposed DB in line with earlier findings [28, 29, 47, 48]. The dense filamentous algae (primarily *Chaetomorpha*) at the base of the eelgrass at site KN may act as an additional substrate for e.g. epiphytic diatoms and harpacticoid copepods [52], as well as refuge for larger epifauna. The algae may thus fuel higher trophic levels by hosting a community with a fast turnover.

Our study showed mesograzers as important components in both seagrass ecosystems, supporting the notion that these play large roles in the regulation of opportunistic algae and, hence, in buffering eutrophication effects in eelgrass beds [50, 98–100]. The biomass of amphipod and isopod mesograzers recorded here (0.15–0.40 g AFDW m^-2^, S1 Table) are similar to mesograzer biomasses in the northern part of Öresund, but 3–8 times smaller than in the northern Baltic Sea [50]. Surprisingly, KN and DB have similar composition, biomass and density of mezograzers, but the higher algal biomass at KN suggests that algal production at this site could be beyond grazer control during parts of the year—a pattern similar to the Swedish west coast [18, 50, 98]. At DB, both exposure and nutrient transport as well as relatively higher grazer biomasses may jointly contribute to more pristine conditions.

We used stable isotopes to identify food sources and consumer linkages in the eelgrass ecosystems (e.g. [49, 61, 101, 102]). The gradual increase in consumer δ^15^N signatures proved valuable for distinguishing primary and secondary consumers [61]. However, the overlapping signatures found for large consumers suggest that many of these species have mixed food sources, being trophic omnivores (Tables 5 and 6). Another interesting finding was that the relatively small gammarid amphipods in KN also feed on the smaller amphipods despite unlimited access to algal food.

There was a striking difference in the diversity and biomass of intermediate predatory fish in DB (12 species, 1.2 g AFDW m^-2^) and KN (6 species, 6.7 g AFDW m^-2^). Reduced fish diversity with eutrophication and associated changes in water clarity (affecting the success of visual predators) has been shown in lakes [103] but not yet in marine ecosystems. This could contribute to the lower fish diversity in KN compared to DB, but the effect is likely minor as shading was not a major problem in these shallow eelgrass beds. The biomass in KN was completely dominated by the three-spined stickleback and the black goby. This is similar to the current situation in most eelgrass meadows along the Swedish west coast, where the biomass of intermediate fish predators has increased ten-fold over 30 years, now reaching a mean of 11 g AFDW m^-2^ [18, 50]. For the Swedish system, it was concluded that the combined effect of overfishing and a 4–8 times increase in nutrient load since the 1930s together favor the abundance of intermediate predators, and thus drive the decline of their food, the mesograzers. Comparisons with studies in KN in the early 1990’s indicate a two to three-fold increase of three-spined sticklebacks and black gobies despite reduced nutrient loading [104]. Both stable isotopes (Tables 3 and 6) and stomach content analysis (S3 Table) showed that the three-spined sticklebacks in KN consume benthic animals, whereas planktonic copepods constitute an important diet in DB. This could indicate a niche differentiation between the abundant jellyfish and three-spined sticklebacks, which limits sticklebacks to benthic food sources in KN [64], where jellyfish effectively control the zooplankton [59].

### Biomass pyramids

A fundamental insight into ecosystem function can be obtained by looking at the energy flow between autotrophs and heterotrophs [38, 105]. The biomass distribution across trophic levels can be a useful measure of food web structure, as it integrates functional properties such as the flow pattern and efficiency of energy transfer as well as turnover rates of different food items [38, 106–108]. Biomass pyramids and the log_10_Biomass vs. trophic level slope are valuable visualizations and tools of energy transfer in ecosystem comparisons [44, 46, 109]. Although informative, we are unaware of any similar approaches of energy transfer estimates in seagrass food webs.

In DB, the biomass structure exhibited a classical pyramidal shape with a negative slope showing an average factor 2.4 decrease between the faunal trophic levels, which indicates a certain delay in energy transfer between the trophic levels. Albeit weaker, the slope in DB suggests some degree of stability [40]. In contrast, there was barely any change in biomass between trophic levels 2–4 in KN, indicating a highly energy-subsidized system with fast turnover of primary producers fueling higher trophic levels and a parallel detritus web [44, 46, 110]. Such high relative biomass at the top of the food chain has been suggested to limit stability through increased strength of top-down interactions [111]. The slope of biomass change with trophic level has been used as an indicator of stability and the relative number of strong vs. weak interaction links in both simple and complex food webs [40, 75, 76, 111]. For example, it has been shown that a slope with a factor 10 decrease will generate a stabilizing pattern [75].

### Species richness and stability

The low fish diversity in KN compared to DB represents one of the most important differences between the two systems. Species richness is one indicator of ecosystem stability, as fewer interactions decrease the robustness to perturbations targeting particular species [40]. This has been demonstrated in other eelgrass ecosystems where robustness was found to decrease at more eutrophied sites [23]. Given the proximity of KN to the ocean, it is unlikely that fish diversity has historically been low. Potentially, overfishing and eutrophication have worked in concert over several decades and changed the ecosystem. Consequently, the system favors omnivorous species and re-establishment of new fish species is hampered by predation and competitive exclusion by jellyfish and intermediate predators [112, 113], along with hypoxia [114] and reduced foraging success due to dominance of filamentous algae [115]. Although KN has been described as an inherently unstable ecosystem [52], the situation today indicates surprisingly little structural change since the reductions in nutrient input [52, 104, 116]. The system appears to remain in a fixed configuration with a high productivity and turnover of both algae and eelgrass and large biomass of consumers. The detrital pathway may maintain the high consumer biomass, and exert an important stabilizing effect by providing a persistent energy supply during times when the energy input from other sources is lower [46, 117, 110].

Ecosystem structure depends on a multitude of environmental factors as discussed above. The present study compared two sites differing in exposure, connection to the open sea and eutrophication at a single point in time. By sampling two nearby sites simultaneously we minimized spatial differences in e.g. climate and potential species pool and minimized effects of seasonal fluctuations in biomass of seagrass and fauna, but cannot account for random variation in these. While our approach allowed us to characterize both ecosystems thoroughly, this high level of detail came at the cost of replication, implying that we are unable to separate the effects of the various regulating factors. Whether our findings of differences in food web structure and trophic level biomass distribution apply to other eelgrass habitats with similar combinations of environmental parameters therefore remains to be tested.

In conclusion, the seagrass meadow and food web structure differed markedly between the two contrasting study sites. Our findings largely support the hypotheses that nutrient-rich, protected settings are characterized by a dominance of opportunistic algae, faster turnover of primary producers and lack of large stationary fish. This results in a simplified food web with high biomass of intermediate predators controlling grazers and further stimulating the proliferation of opportunistic algae, relative to more pristine and exposed settings. These results suggest that the physical setting of seagrass meadows influences ecosystem structure, function and resilience. We hope that the current study will inspire further initiatives in this direction and contribute to build a data base allowing further generalizations than the current survey of two contrasting ecosystems can underpin.

## Supporting Information

S1 Table Abundance and biomass of infauna and epifauna collected from Dalby Bay (DB) and Kertinge Nor (KN) in Denmark, June 2011. N = 6, except epifauna in DB (n = 7). Total mean ± SE is calculated from sample totals. Biomass in ash-free dry weight (AFDW) was calculated by conversion factors from: abundance of epifauna passing a 1mm sieve, wet weight of epifauna retained on a 1mm sieve, and dry weight of infauna—see materials and methods, and raw data in DRYAD.
(XLSX)

S2 Table Abundance and biomass of fish in Dalby Bay (DB) and Kertinge Nor (KN) in Denmark, June 2011. Fish abundance and biomass was multiplied by 3.5 due to underestimation of the beach seine methods. Area estimates were based on one haul covering 250 m2. Ash-free dry weight (AFDW) were calculated from wet weight by conversion factors (see materials and methods).
(XLSX)

S3 Table Stomach content analysis of fish species in Dalby Bay (DB) and Kertinge Nor (KN) in Denmark, June 2011. FO% = Frequency of occurrence in % of total number of stomachs.
# = mean abundance in stomachs containing the prey item ± SD. Size range of prey items provided for *G*. *aculeatus* in Kertinge Nor.
(XLSX)

S4 Table Proportion of faunal biomass assigned to trophic levels (TL), and sources for the assignment. (XLSX)
